# Supplementary material for: Single Cell-Type Spatial Proteomics Uncovers Regional Heterogeneity of Astrocytes
Source: J Proteome Res. 2026 Jul 17;25(8):3826–34. doi: 10.1021/acs.jproteome.6c00321 (PMC13459543; doi:10.1021/acs.jproteome.6c00321)
Supplement: Supplementary file 1 [file pr6c00321_si_001.pdf]

# Single Cell-Type Spatial Proteomics Uncovers Regional Heterogeneity of Astrocytes

Chien-Chang Huang[1], Chiung-Yun Chang[1], Po-Chao Chan[1], Weng Man Chong[1], Hsiao-Jen Chang[1], Jung-Chi Liao[1]*

[1] Syncell Inc., Taipei, 115202, Taiwan

*E-mail: jcliao@syncell.com

Figure S1: Validation of a cortical astrocyte candidate LAMC3 via confocal microscopy.

Table S1: List of antibodies used for immunofluorescent staining.

Table S2: Raw proteome dataset for cortical/hippocampal astrocytes (XLSX).

Table S3: Comprehensive proteome of cortical astrocytes (XLSX).

Table S4: Comprehensive proteome of hippocampal astrocytes (XLSX).

Table S5: Curated database of canonical astrocyte marker proteins (XLSX).

Table S6: Commonly identified proteins across two cortical astrocyte replicates (XLSX).

Table S7: Commonly identified proteins across two hippocampal astrocyte replicates (XLSX).

Table S8: Proteins enriched specifically in cortical astrocytes (XLSX).

Table S9: Proteins enriched specifically in hippocampal astrocytes (XLSX).

# Supplementary Figure S1

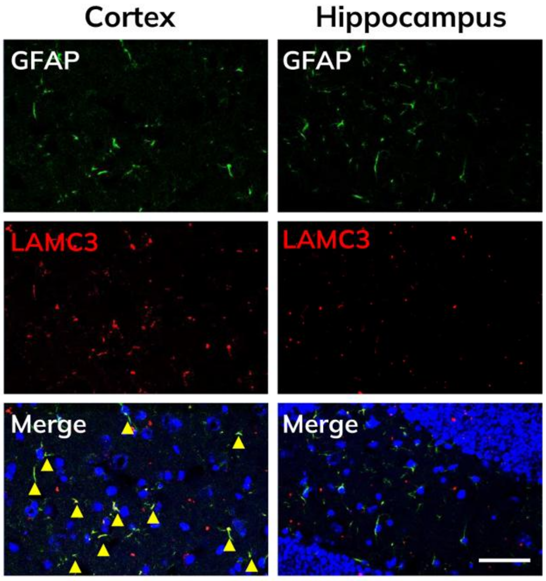


Supplementary Figure S1. Validation of a cortical astrocyte candidate LAMC3 via confocal microscopy. Representative confocal images showed the expression and localization of LAMC3 within cortical and hippocampal astrocytes. Astrocytes were identified by co-staining with the canonical marker GFAP (green) and DAPI for nuclei (blue). Yellow arrowheads indicate the colocalization of GFAP with LAMC3. Scale bar, 50 μm.

**Supplementary Table 1. List of antibodies used for immunofluorescent staining.**

| Antibodies | Source | Identifier |
|---|---|---|
| Rabbit polyclonal anti-COTL1 | Proteintech | Cat# 10781-1-AP, RRID: AB_2084785 |
| Rabbit polyclonal anti-CDC42 | Proteintech | Cat# 10155-1-AP, RRID: AB_2078096 |
| Rabbit polyclonal anti-SLC6A11 | Proteintech | Cat# 13920-1-AP, RRID: AB_2877988 |
| Rabbit polyclonal anti-RPL8 | Proteintech | Cat# 16981-1-AP, RRID: AB_2182071 |
| Rabbit polyclonal anti-PSD3 | Novus | Cat# NBP2-55678, RRID: AB_3340881 |
| Rabbit polyclonal anti-Macro H2A.2 | GeneTex | Cat# GTX116234, RRID: AB_10621251 |
| Rabbit polyclonal anti-CORO1C | Proteintech | Cat# 14749-1-AP, RRID: AB_2230110 |
| Rabbit polyclonal anti-GLUT3 | Proteintech | Cat# 20403-1-AP, RRID: AB_10694437 |
| Rabbit polyclonal anti-Destrin | GeneTex | Cat# GTX11072, RRID: AB_381235 |
| Rabbit polyclonal anti-MINK1 | Proteintech | Cat# 13137-1-AP, RRID: AB_10644151 |
| Rabbit monoclonal anti-AP2S1 | Proteintech | Cat# 84174-3-RR, RRID: AB_3671731 |
| Rabbit polyclonal anti-HRAS | Proteintech | Cat# 18295-1-AP, RRID: AB_2121046 |
| Rabbit polyclonal anti-MINK1 | Proteintech | Cat# 13137-1-AP, RRID: AB_106044151 |
| Rabbit polyclonal anti-PARK7 /DJ1 | Proteintech | Cat# 11681-1-AP, RRID: AB_2160099 |
| Mouse monoclonal anti-LAMC3 | Proteintech | Cat# 67261-1-Ig, RRID: AB_2882532 |
| Rabbit polyclonal anti-Cystatin C | Proteintech | Cat# 12245-1-AP, RRID: AB_2088058 |
| Rabbit polyclonal anti-PLEKHB1 | Proteintech | Cat# 10512-1-AP, RRID: AB_2166943 |
| Rabbit polyclonal anti-FBLN5 | Proteintech | Cat# 12188-1-AP, RRID: AB_2105939 |

| | | |
|---|---|---|
| Rabbit polyclonal anti-Kindlin2 | Proteintech | Cat# 11453-1-AP,<br>RRID: AB_2262660 |

| **Antibodies** | Source | Identifier |
|---|---|---|
| Rabbit polyclonal anti-Rab21 | Proteintech | Cat# 84776-2-RR,<br>RRID: AB_3714719 |
| Rabbit polyclonal anti-GLTP | Proteintech | Cat# 10850-1-AP,<br>RRID: AB_2294627 |
| Rabbit polyclonal anti-Septin4 | Proteintech | Cat# 12476-1-AP,<br>RRID: AB_2187151 |
| Rabbit polyclonal anti-STXBP3 | Proteintech | Cat# 13764-1-AP,<br>RRID: AB_2196832 |
| Rabbit polyclonal anti-C1qC | Proteintech | Cat# 16889-1-AP,<br>RRID: AB_2878328 |
| Mouse monoclonal anti-CNTN2 | Proteintech | Cat# 67089-1-IG,<br>RRID: AB_2918481 |
| Rabbit polyclonal anti-LGI3 | Proteintech | Cat# 21919-1-AP,<br>RRID: AB_10838807 |
| Rabbit polyclonal anti-GFAP | Proteintech | Cat# 81063-1-RR,<br>RRID: AB_2923699 |
| Mouse monoclonal anti-GFAP | Merck Millipore | Cat# MAB360,<br>RRID: AB_11212597 |
| Donkey anti-Rabbit IgG,<br>Alexa Fluor Plus 555 | Thermo Fisher Scientific | Cat# A32794,<br>RRID: AB_2762834 |
| Donkey Anti-Mouse IgG,<br>Alexa Fluor 568 | Thermo Fisher Scientific | Cat# A10037,<br>RRID: AB_11180865 |
| Donkey anti-Rabbit IgG,<br>Alexa Fluor Plus 647 | Thermo Fisher Scientific | Cat# A32795TR,<br>RRID: AB_2866496 |
| Donkey anti-mouse IgG,<br>Alexa Fluor 647 | Thermo Fisher Scientific | Cat# A-21235,<br>RRID: AB_2535804 |
